# Supplementary material for: Genetic influences on hub connectivity of the human connectome
Source: Nat Commun. 2021 Jul 9;12:4237. doi: 10.1038/s41467-021-24306-2 (PMC8271018; doi:10.1038/s41467-021-24306-2)
Supplement: Supplementary file 2 — Reporting Summary [file 41467_2021_24306_MOESM2_ESM.pdf]

nature research

Corresponding author(s): Aurina Arnatkeviciute

Last updated by author(s): 2021/04/29

Reporting Summary

Nature Research wishes to improve the reproducibility of the work that we publish. This form provides structure for consistency and transparency in reporting. For further information on Nature Research policies, see our Editorial Policies and the Editorial Policy Checklist.

Statistics

For all statistical analyses, confirm that the following items are present in the figure legend, table legend, main text, or Methods section.

n/a	Confirmed	
☐	☒	The exact sample size (*n*) for each experimental group/condition, given as a discrete number and unit of measurement
☐	☒	A statement on whether measurements were taken from distinct samples or whether the same sample was measured repeatedly
☐	☒	The statistical test(s) used AND whether they are one- or two-sided<br>*Only common tests should be described solely by name; describe more complex techniques in the Methods section.*
☐	☒	A description of all covariates tested
☐	☒	A description of any assumptions or corrections, such as tests of normality and adjustment for multiple comparisons
☐	☒	A full description of the statistical parameters including central tendency (e.g. means) or other basic estimates (e.g. regression coefficient) AND variation (e.g. standard deviation) or associated estimates of uncertainty (e.g. confidence intervals)
☐	☒	For null hypothesis testing, the test statistic (e.g. *F*, *t*, *r*) with confidence intervals, effect sizes, degrees of freedom and *P* value noted<br>*Give P values as exact values whenever suitable.*
☒	☐	For Bayesian analysis, information on the choice of priors and Markov chain Monte Carlo settings
☐	☒	For hierarchical and complex designs, identification of the appropriate level for tests and full reporting of outcomes
☐	☒	Estimates of effect sizes (e.g. Cohen's *d*, Pearson's *r*), indicating how they were calculated

Our web collection on statistics for biologists contains articles on many of the points above.

Software and code

Policy information about availability of computer code

Data collection	Details for the publicly available data used in this analysis can be found at https://db.humanconnectome.org/ (Human Connectome project), https://human.brain-map.org/ (Allen Human Brain atlas) and https://bigbrainproject.org/ (BigBrain project).
Data analysis	The code to reproduce the figures: is provided in the github repository: https://github.com/BMHLab/GeneticBrainHubs.git. Software packages used include: MATLAB_R2019b, FreeSurfer v5.3.0, FSL version 5.0.11, MRtrix3 3.0.15 (iFOD2 algorithm used for tractography), FMRIB Software Library (5.0.9 for HCP data and 5.0.11 for Monash sample), OpenMx 2.15, in R (3.5.3), Re-Annotator, fast_marching_toolbox, ErmineJ version 3.2.

For manuscripts utilizing custom algorithms or software that are central to the research but not yet described in published literature, software must be made available to editors and reviewers. We strongly encourage code deposition in a community repository (e.g. GitHub). See the Nature Research guidelines for submitting code & software for further information.

Data

Policy information about availability of data

All manuscripts must include a data availability statement. This statement should provide the following information, where applicable:

- Accession codes, unique identifiers, or web links for publicly available datasets
- A list of figures that have associated raw data
- A description of any restrictions on data availability

Structural brain networks were mapped from Human Connectome Project data (https://db.humanconnectome.org/).
Gene expression data were acquired from the Allen Human Brain Atlas (https://human.brain-map.org/static/download).
Microstructural profiles were derived from the BigBrain Project (https://bigbrainproject.org/).
Gene ontology annotations were obtained from GEMMA https://gemma.msl.ubc.ca/ arrays/showArrayDesign.html?id=735 as

Generic_human_ncbiIds_noParents.an.txt.gz on April 29, 2021.
Gene Ontology terms and definitions were automatically downloaded by ErmineJ on April 29, 2021 as go.obo (data version 2021-02-01) and can be downloaded from http://release.geneontology.org/2021-02-01/ontology/index.html.
Custom MATLAB code, and other data necessary to generate the figures in this work are available at https://github.com/BMHLab/GeneticBrainHubs and associated repository https://doi.org/10.5281/zenodo.4724186 respectively.

Field-specific reporting

Please select the one below that is the best fit for your research. If you are not sure, read the appropriate sections before making your selection.

☒ Life sciences ☐ Behavioural & social sciences ☐ Ecological, evolutionary & environmental sciences

For a reference copy of the document with all sections, see nature.com/documents/nr-reporting-summary-flat.pdf

Life sciences study design

All studies must disclose on these points even when the disclosure is negative.

Sample size	All available data surpassing the quality control criteria were used in each analysis. Group-representative connectome was generated based on the 972 subjects from the Human Connectome project; Heritability analysis included 117 pairs of genetically confirmed monozygotic (MZ) twin pairs and 69 of their non-twin siblings, as well as 60 dizygotic (DZ) same-sex twin pairs and 48 of their non-twin siblings. Group-representative connectome for Monash sample was generated based on the data from 424 subjects.
Data exclusions	Subjects from both DWI datasets were excluded based insufficient connectome density (more than 3SD below the mean of the sample).<br>5 subjects from Monash sample were excluded due to issues with cortical surface segmentation.
Replication	We replicated the main heritability and transcriptional coupling results using different data processing choices (brain parcellations and connectome densities) by applying the same analysis to data derived from 2 brain parcellations (180 and 250 regions per hemisphere respectively) and across three connectome density thresholds. Across all iterations connections between hub regions (rich links) were more heritable compared to other link types (results presented in Figure S4). For transcriptional coupling analysis, connected pairs of hubs demonstrated increased transcriptional coupling (results presented in Figure S7). The results demonstrating the increase in microstructural profile covariance were not replicated using random parcellation containing 500 regions. This discrepancy likely reflects the fact that the HCPMMP1 parcellation more closely approximates boundaries between functional zones of the cortex, as it is based on a fusion of multimodal imaging data. The random parcellation makes no attempt to capture such boundaries and may blur different cytoarchitectonic regions within the same network node, thus resulting in noisier microstructural profile covariance (MPC) estimates. In this way, the MPC results appear to depend on accurate approximation of cytoarchitectonic boundaries in cortex.
Randomization	No experimental conditions requiring randomization were applied in the study.
Blinding	No procedures requiring blinding were applied in the study.

Reporting for specific materials, systems and methods

We require information from authors about some types of materials, experimental systems and methods used in many studies. Here, indicate whether each material, system or method listed is relevant to your study. If you are not sure if a list item applies to your research, read the appropriate section before selecting a response.

Materials & experimental systems

n/a	Involved in the study	
☒	☐	Antibodies
☒	☐	Eukaryotic cell lines
☒	☐	Palaeontology and archaeology
☒	☐	Animals and other organisms
☐	☒	Human research participants
☒	☐	Clinical data
☒	☐	Dual use research of concern

Methods

n/a	Involved in the study	
☒	☐	ChIP-seq
☒	☐	Flow cytometry
☐	☒	MRI-based neuroimaging

Human research participants

Policy information about studies involving human research participants

Population characteristics	HCP dataset: 972 subjects, age (mean) = 28.7 (SD=3.7), 522 females. Heritability analyses included sex and age as covariates. Monash dataset: 424 subjects, age (mean) = 23.5 (SD=5.3), 190 females.
Recruitment	Participants for the Monash Sample were recruited through online advertisements. The sample is likely to include a relatively high number of university students which is representative of healthy young population that was required for this study and should not impact the results of the study. Details regarding the recruitment process of the HCP dataset is provided in the following paper: https://doi.org/10.1016/j.neuroimage.2013.05.041/. In short, the sample includes healthy adults with no neuropsychiatric or neurological disorders including siblings and twin pairs. Twins born prior to 34 weeks gestation and non-twins born prior to 37 weeks gestation are excluded in order to avoid potential confounding factors. Subject selection procedure should not influence the results of this study.
Ethics oversight	The experimental protocol was approved by Monash University's Human Research Ethics Committee and was carried out in accordance with the approved guidelines. Informed consent was obtained from all participants before testing. For HCP dataset subject recruitment procedures and informed consent forms, including consent to share de-identified data, were approved by the Washington University institutional review board. Full details can be found in Glasser et. al., 2013.

Note that full information on the approval of the study protocol must also be provided in the manuscript.

Magnetic resonance imaging

Experimental design

Design type	Diffusion and structural scans
Design specifications	Diffusion and structural scans were collected at rest.
Behavioral performance measures	Behavioural measures were not used in this study

Acquisition

Imaging type(s)	structural, diffusion
Field strength	3T
Sequence & imaging parameters	HCP: Data were acquired on a customized Siemens 3T "Connectome Skyra" scanner at Washington University in St Louis, Missouri, USA using a multi-shell protocol for the DWI: 1.25mm3 isotropic voxels, repetition time (TR) = 5520 ms, echo time (TE) = 89.5 ms, field-of-view (FOV) of 210 × 180 mm, 270 directions with b = 1000, 2000, 3000 s/mm2 (90 per b value), and 18 b = 0 volumes. Structural T1-weighted data were collected using 0.7mm3 isotropic voxels, TR = 2400 ms, TE = 2.14 ms, FOV of 224 × 224 mm.<br>Monash Sample: data obtained on a Siemens Skyra 3T scanner at Monash Biomedical Imaging in Clayton, Victoria, Australia using the following parameters: 2.5mm3 voxel size, TR = 8800 ms, TE = 110 ms, FOV of 240 × 240 mm, 60 directions with b = 3000 s/mm2 and seven b = 0 volumes. In addition, a single b = 0 s/mm2 was obtained with the reverse-phase encoding so distortion correction could be performed. T1-weighted structural scans were acquired using: 1mm3 isotropic voxels, TR = 2300 ms, TE = 2.07 ms, FOV of 256 × 256 mm.
Area of acquisition	Whole brain

Diffusion MRI ☒ Used ☐ Not used

Parameters: HCP: 270 directions with b = 1000, 2000, 3000 s/mm2 (90 per b value), and 18 b = 0 volumes.
Monash Sample: 60 directions with b = 3000 s/mm2 and seven b = 0 volumes.

Preprocessing

Preprocessing software	FreeSurfer v5.3.0 software, FSL version 5.0.11, MRtrix3 3.0.15.
Normalization	HCP: normalization of mean b0 image across diffusion acquisitions.
Normalization template	Connectomes reconstructed in subject-specific spaces.
Noise and artifact removal	HCP: correction for EPI susceptibility and signal outliers, eddy-current-induced distortions, slice dropouts, gradient nonlinearities and subject motion.<br>Monash Sample: Distortions in the Monash DWI data were corrected with TOPUP in FSL, using the forward and reverse phase-encoded b = 0 images to estimate the susceptibility-induced off-resonance field. We corrected for eddy-current distortions, volume-to-volume head motion, within-volume head motion, and signal outliers using eddy tool in FSL [version 5.0.11]. This implementation of EDDY significantly mitigates motion-related contamination of DWI connectivity estimates. DWI data were subsequently corrected for B1 field inhomogeneities using FAST in FSL.

Volume censoring	The processing of structural and diffusion scans do not require volume censoring.

Statistical modeling & inference

Model type and settings	Structural equation modelling for heritability analysis. Correlational analysis for gene expression analysis. Mathematical modelling with parameter optimization via 2D Voronoi partitioning method for modelling analysis.
Effect(s) tested	Phenotypic variance explained by genetic and environmental effects quantified with structural equation modelling. Differences in correlated gene expression estimates tested for gene expression analysis. Fits to network topological distributions evaluated in modelling analysis.

Specify type of analysis: ☒ Whole brain ☐ ROI-based ☐ Both

Statistic type for inference (See Eklund et al. 2016)	Connectome-wide, with effects quantified at each connection in whole-brain networks.
Correction	FDR correction for multiple comparisons was applied in GO enrichment analysis.

Models & analysis

n/a	Involved in the study
☒	☐ Functional and/or effective connectivity
☐	☒ Graph analysis
☒	☐ Multivariate modeling or predictive analysis

Graph analysis	Weighted and binary rich-club coefficients, weighted and binary edge communicability, pairwise structural connectivity
